# Supplementary material for: Identification of Freezing-Responsive microRNAs and Their Targets in Chinese Jujube by Small RNA and Degradome Sequencing
Source: Int J Mol Sci. 2026 Sep 4;27(17):7912. doi: 10.3390/ijms27177912 (PMC13566890; doi:10.3390/ijms27177912)
Supplement: Supplementary file 1 [file ijms-27-07912-s001.zip › Supplementary Material S2/novel_miR_173.pdf]

Provisional ID	:	novel_miR_173
Score total	:	0.4
Score for star read(s)	:	-1.3
Score for read counts	:	0
Score for mfe	:	0.1
Score for randfold	:	1.6
Score for cons. seed	:	
Total read count	:	456
Mature read count	:	456
Loop read count	:	0
Star read count	:	0

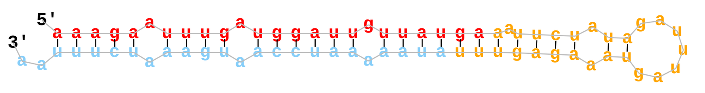

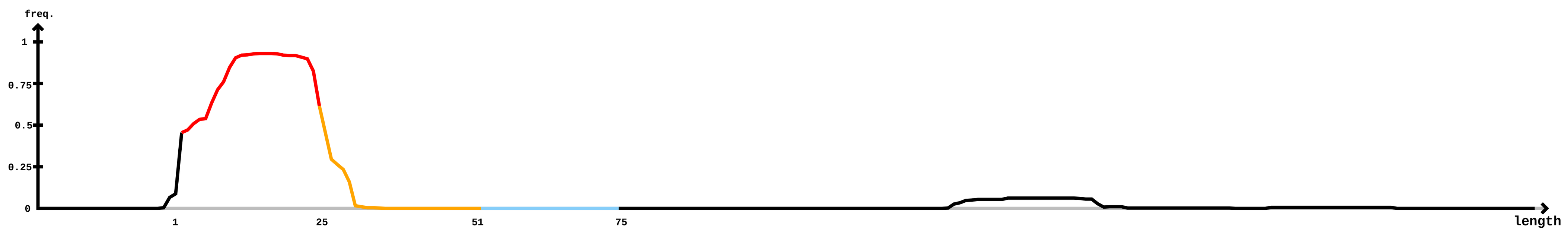

Mature   Star

```
5'- auuuaaaaugaguuauaauuuuaaagaauuugauggauuguuaugaaauucuauagauuuaguaaagaguuuauaaaaaauccaaugaaaucuuuaagauuaaugcuggauuucauauuaauaauuuucuucacaauuucacuauugaaauccuuucaaauccauuaaaauccaucauuuuuuaaaaucuuuuaaaauuaaugauuuuuuuaauaucauuacauuuuaaaagaauuuuacaaaauccuuau  -3'   exp
    ..........(((..((((((((((((.((((.((((((.(((((((.((((.((.......)).))))))))))).)))))).)))).))))))))))))..)))((((((((.......((((........)))).......))))))))......................(((((.((((((((((((((((((((((.........))))))).))))))))))).)))))).)))))......   reads   mm   sample
    ...................uuuaaagaauuugauggau        1   0   LT-1
    ...................uuuaaagaauuugauggauu       1   0   JCK-2
    ....................uuaaagaauuugauggauu       1   0   JCK-1
    ....................uuaaagaauuugauggauu       2   0   LT-2
    ....................uuaaagaauuugauggauug      1   0   LT-2
    ....................uuaaagaauuugauggauuguua   2   0   JCK-1
    ....................uuaaagaauuugauggauuguua   1   0   LT-2
    ....................uuaaagaauuugauggauuguua   1   0   JCK-3
    ....................uuaaagaauuugauggauuguuau  3   0   JCK-1
    ....................Cuaaagaauuugauggauuguuau  2   1   JCK-1
    ....................uuaaagaauuugauggauuguuau  5   0   JCK-3
    ....................uuaaagaauuugauggauuguAau  1   1   JCK-1
    ....................uuaaagaauuugauggauuguuau  4   0   LT-1
    ....................uuaaagaauuugaugAauuguuau  2   1   LT-1
    ....................uuaaagaauuugauggauuguCau  1   1   JCK-3
    ....................uuaaagaauuugauggauuguuGu  1   1   JCK-2
    ....................uuaaagaauuugauggauuguuau  3   0   JCK-2
    ....................uuaaagaauuugauggauuguuau  1   0   LT-3
    .....................Aaaagaauuugauggauuguuaug  1   1   LT-2
    .....................Aaaagaauuugauggauuguuaug  2   1   LT-3
    .....................Aaaagaauuugauggauuguuaug  2   1   JCK-3
    .....................Aaaagaauuugauggauuguuaug  3   1   LT-1
    .....................Aaaagaauuugauggauuguuaug  2   1   JCK-2
    .....................Aaaagaauuugauggauuguuauga 1   1   JCK-1
    ......................aaagaauuugauggauuguA     4   1   LT-2
    ......................aaagaauuugauggauuguu     1   0   LT-2
    ......................aaagaauuugauggauuguuau   2   0   JT-2
    ......................aaagaauuugauggauuguuau   2   0   JCK-3
    ......................aaagaauuugauggauuguuau   5   0   JT-3
    ......................aaagaauuugauggauuguuaA   2   1   LT-3
    ......................aaagaauuugauggauuguuau   1   0   JCK-1
    ......................aaagaauuugauggauuguuau   2   0   JCK-2
    ......................aaagUauuugauggauuguuaug  4   1   JCK-2
    ......................aaagaauuugauggaCuguuaug  1   1   JCK-1
```

```
                      Mature                                                Star
auuuaaaaugaguuauaauuuuaaagaauuugauggauuguuaugaaauucuauagauuuaguaaagaguuuauaaaaauccaaugaaaucuuuaagauuaaugcuggauuucauauuaauaauuuucuucacaauuucacuauugaaauccuuucaaauccauuaaaauccaucauuuuuuaaaaucuuuuaaaauuaaugauuuuuuuaauaucauuacauuuuaaaagaauuuuacaaaauccuuau
......................aaagaauuugauggauuguAaug.............................................................................................................................................................................................................   1   1   LT-1
......................aaagUauuugauggauuguuaug.............................................................................................................................................................................................................   3   1   LT-2
......................aaagaauuugauggauuguuaug.............................................................................................................................................................................................................   13  0   JCK-2
......................aaagUauuugauggauuguuaug.............................................................................................................................................................................................................   2   1   JCK-1
......................aaagaauuugauggauuguuaug.............................................................................................................................................................................................................   9   0   JCK-3
......................aaagUauuugauggauuguuaug.............................................................................................................................................................................................................   1   1   JCK-3
......................aaagaauuugauggaCuguuaug.............................................................................................................................................................................................................   2   1   JCK-2
......................aaagaauuugauggauuAuuaug.............................................................................................................................................................................................................   1   1   LT-2
......................aaagaauuugauggauuguuaug.............................................................................................................................................................................................................   9   0   LT-2
......................aaagaauuugauggauuguuaug.............................................................................................................................................................................................................   11  0   JCK-1
......................aaagaauuugauggauuguuaug.............................................................................................................................................................................................................   2   0   LT-1
......................aaagaauuugauggauuguuauA.............................................................................................................................................................................................................   2   1   JCK-1
......................aaagaauuugauggauuguuaug.............................................................................................................................................................................................................   4   0   LT-3
......................aaagaauuugauggaCuguuaug.............................................................................................................................................................................................................   1   1   JCK-3
......................aaagaauuugauggauuguuauga............................................................................................................................................................................................................   20  0   JCK-1
......................aaagaauuugauggauuguuauga............................................................................................................................................................................................................   6   0   LT-1
......................Gaagaauuugauggauuguuauga............................................................................................................................................................................................................   1   1   JCK-3
......................aaagaauuugauggauuguuauga............................................................................................................................................................................................................   2   0   LCK-3
......................Gaagaauuugauggauuguuauga............................................................................................................................................................................................................   3   1   JCK-2
......................aaagaauuugauggauuguuauga............................................................................................................................................................................................................   23  0   JCK-3
......................aaagaauuugauggauuguuauga............................................................................................................................................................................................................   5   0   LT-3
......................aaagaauuugauggauuguuauga............................................................................................................................................................................................................   2   0   LT-2
......................aaagaauuugauggauuguuauga............................................................................................................................................................................................................   35  0   JCK-2
......................aaagaauuugauAgauuguuauga............................................................................................................................................................................................................   2   1   JCK-2
.......................aagaauuugauggauuguuaug.............................................................................................................................................................................................................   3   0   LCK-3
.......................aagaauuugauggauuguuauga............................................................................................................................................................................................................   2   0   JCK-3
.......................aagaauuugauggauuguuauga............................................................................................................................................................................................................   2   0   LT-1
.......................aaAaauuugauggauuguuaugaa...........................................................................................................................................................................................................   1   1   JCK-2
........................agaGuuugauggauuguua...............................................................................................................................................................................................................   1   1   LT-1
........................agaauuugauggaCuguuaug.............................................................................................................................................................................................................   1   1   JCK-3
........................agaauuugauggauuguuaug.............................................................................................................................................................................................................   1   0   LT-1
........................agaGuuugauggauuguuaug.............................................................................................................................................................................................................   1   1   JCK-2
........................agaauuugauggauuguuaug.............................................................................................................................................................................................................   6   0   LCK-3
........................agaGuuugauggauuguuaug.............................................................................................................................................................................................................   1   1   JCK-3
........................agaauuugauggauuAuuauga............................................................................................................................................................................................................   4   1   LCK-3
........................agaauuugauAgauuguuaugaa...........................................................................................................................................................................................................   1   1   LT-3
........................agaauuugauggauuguuaugaa...........................................................................................................................................................................................................   1   0   LT-3
........................agaauuugauggauuguuaugaa...........................................................................................................................................................................................................   2   0   JCK-2
.........................gaauuugauggauuguuaug.............................................................................................................................................................................................................   1   0   JCK-2
.........................gaauuugauggauuguuaug.............................................................................................................................................................................................................   3   0   JCK-3
.........................gaauuugauggauuguuaCg.............................................................................................................................................................................................................   1   1   JCK-1
.........................gaauuugauggauuguuaug.............................................................................................................................................................................................................   1   0   LT-3
.........................gaauuugauggauuguuauA.............................................................................................................................................................................................................   1   1   LT-2
.........................gaauuugauggauuguuauC.............................................................................................................................................................................................................   1   1   JCK-1
.........................gaauuugauggauuguuaCg.............................................................................................................................................................................................................   1   1   JCK-2
.........................gaauuugauggauuguuaug.............................................................................................................................................................................................................   1   0   LT-1
.........................gaauuugauggauuguuauga............................................................................................................................................................................................................   1   0   LT-1
.........................Aaauuugauggauuguuauga............................................................................................................................................................................................................   1   1   JCK-2
.........................gaauuugauggauuguuaugaaau.........................................................................................................................................................................................................   1   0   JCK-1
..........................aauuugauggauuguuauga............................................................................................................................................................................................................   2   0   LT-1
...........................auuugauggauuguuaug.............................................................................................................................................................................................................   1   0   LT-2
...........................auuugauggauuguuaug.............................................................................................................................................................................................................   3   0   LT-3
...........................auuugauggauuguuaug.............................................................................................................................................................................................................   1   0   JCK-1
...........................auuugauggauuguuauU.............................................................................................................................................................................................................   1   1   LT-3
...........................Uuuugauggauuguuauga............................................................................................................................................................................................................   1   1   LT-3
...........................auuugauggauuguuauga............................................................................................................................................................................................................   6   0   JCK-2
...........................auuugauggauuguuauga............................................................................................................................................................................................................   3   0   LT-3
...........................auuugauggauuguuauga............................................................................................................................................................................................................   4   0   LCK-3
...........................auuugauAgauuguuauga............................................................................................................................................................................................................   1   1   LT-2
...........................auuugauggauuguuaugUa...........................................................................................................................................................................................................   1   1   LT-3
...........................auuugauggauuguuaugaU...........................................................................................................................................................................................................   1   1   JCK-3
...........................auuugauggauuguuaAgaa...........................................................................................................................................................................................................   1   1   LT-3
...........................auuugauggauuguuaugaaa..........................................................................................................................................................................................................   1   0   LT-1
...........................auuugauggauuguuauAaaau.........................................................................................................................................................................................................   7   1   JT-3
...........................auuugauggauuguuaugaaau.........................................................................................................................................................................................................   3   0   JCK-3
...........................auuugauggauuguuaugaaau.........................................................................................................................................................................................................   1   0   JCK-1
...........................auuugauggauuguuaugaaau.........................................................................................................................................................................................................   2   0   JCK-2
...........................auuugauggauuguuaugUaauu........................................................................................................................................................................................................   1   1   JCK-2
...........................auuugauggauuguuaugUaauu........................................................................................................................................................................................................   1   1   JCK-1
...........................auuugauggauuguuaugaaauu........................................................................................................................................................................................................   1   0   JCK-1
```

Mature **Star**

auuuaaaaugaguuauaauuuu**aaagaauuugauggauuguuauga**aauucuauagauuuaguaaagaguuu**auaaaaauccaaugaaaucuuuaa**gauuaaugcuggauuucauauuaauaauuuucuucacaauuucacuauugaaauccuuucaaauccauuaaaauccaucauuuuuuaaaaucuuuuaaaauuaaugauuuuuuuaauaucauuacauuuuaaaagaauuuuacaaaauccuuau

auuugauggauuguuauAaaauu	1	1	JCK-2
auuugauggauuguuaugaaauu	1	0	JCK-2
auuugauggauuguuaugaaauu	2	0	LT-3
auuugauggauuguuaugaaauu	2	0	JCK-3
uuugauggauuguuauga	2	0	LT-1
uuugaAggauuguuauga	4	1	LT-2
uuugauggauuguuaugaa	13	0	LCK-1
uuugauggauuguuauUaa	1	1	LT-2
uuugauggauuguuaugaa	5	0	LCK-2
uuugauggauuguuaugaaau	1	0	JCK-1
uuugauggauuguuaugaaauu	2	0	JCK-2
uuugauggauuguuaugUaauuc	6	1	LCK-3
uuugauggauuguuaugaaauAc	1	1	JCK-3
uuugauggauuguuaugaaauuc	1	0	JCK-1
uuugauggauuguuaugaaauuc	3	0	JCK-2
uuugauggauuguuaugaaauAc	1	1	JCK-1
uugauggauuguuaugaaauu	2	0	LT-3
uugauggauuguuaugaaauu	1	0	JCK-3
uugauggauuguuaugaaUuu	3	1	LT-2
uugauggauuguuaugaaauu	1	0	JCK-1
uugauggauuguAaugaaauu	5	1	LCK-3
uugauggauuguuaugaaauu	1	0	JCK-2
uugauggauuguuaugaaauuc	8	0	LT-2
uugauggauuguAaugaaauuc	2	1	LT-2
uugauggauuguuaugaaauuc	1	0	JCK-1
ugauggauuguAaugaaa	6	1	JT-2
ugauggauuguAaugaaa	9	1	JT-3
uUauggauuguuaugaaauu	3	1	LCK-3
ugauAgauuguuaugaaauu	1	1	LT-1
Cgauggauuguuaugaaauu	1	1	LT-1
ugauggauuguuaugaaauuc	2	0	LT-3
ugauggauuguuaugaaauuc	4	0	LT-2
ugauggauuguuaugaaauuc	1	0	JCK-3
ugauggauuguuaugUaauuc	1	1	LT-1
ugauggauuguuaugaaauuc	9	0	LCK-3
uUauggauuguuaugaaauuc	3	1	LT-2
ugauggauuguuaugaaauuc	2	0	JCK-1
ugauggauuguuaugaaUuucu	1	1	JCK-1
gauggauugAuaugaaauu	1	1	JCK-1
gauggauugAuaugaaauu	1	1	JCK-2
Aauggauuguuaugaaauu	2	1	JCK-3
Aauggauuguuaugaaauu	2	1	JCK-1
gauggauuguuaugaaauuc	2	0	JCK-3
gauggauuguuauAaaauuc	1	1	JCK-2
gauggauuguuaugaaauuc	2	0	JT-2
gauggauuguuauAaaauuc	1	1	JCK-3
gauggauuguuaugaaauuc	4	0	JCK-2
gauggauuguuaugaaauuc	10	0	LCK-3
gauggauuguuaugaaauuc	3	0	JT-3
auggauuguuaugaaauu	1	0	LT-1
auggauuguuaugaaUuu	1	1	LT-3
auggauuguuaugaaauuc	2	0	LCK-3
auggauuguAaugaaauuc	2	1	LT-2
auAgauuguuaugaaauucu	1	1	LT-1
auggauuguuaugaaauuAu	1	1	LT-1
uggauuguuaugaaauuUuaua	1	1	LT-2
ggauuguuaugaaauucua	3	0	LT-2
gauGguuaugaaauucuauag	1	1	LT-3
ccuuucaaauccauuaaaaucc	1	0	LT-3
cuuucaaauccauuaaaaucca	2	0	LT-3
cuuucaCauccauuaaaauccauc	1	1	JCK-3
cuuucaaauccauuaaaauccauc	1	0	JCK-3
cuuucaaauccauuaaaauccauc	2	0	JCK-2
cuuucaaauccauuaaaauccauc	5	0	JCK-1
Auuucaaauccauuaaaauccauca	1	1	LT-1
uuucaaauccauuaaaauccauc	1	0	JCK-1
uuucaCauccauuaaaauccauc	1	1	JCK-1
uuucaaauccauuaaaauccauca	1	0	JCK-2
uuucaaauccauuaaaauccauca	1	0	JCK-3
uucaaauccauuaaaauccauc	1	0	LT-1

```
                      Mature                                               Star
auuuaaaaugaguuauaauuuuaaagaauuugauggauuguuaugaaauucuauagauuuaguaaagaguuuauaaaaaauccaaugaaaucuuuaagauuaaugcuggauuucauauuaauaauuuucuucacaauuucacuauugaaauccuuucaaauccauuaaaauccaucauuuuuuaaaaucuuuuaaaauuaaugauuuuuuuaauaucauuacauuuuaaaagaauuuuacaaaauccuuau
..........................................................................................................................................................uucaaauccauuaaaauccauc...........................................................................   2   0   JCK-2
..........................................................................................................................................................uucaaauccauuaaaauccauca..........................................................................   1   0   JCK-2
..........................................................................................................................................................uucaaauccauuaaaauccauca..........................................................................   1   0   LT-2
..........................................................................................................................................................uucaaauccauuaaaauccauca..........................................................................   1   0   LT-1
..........................................................................................................................................................uucaaauccauuaaaauccauca..........................................................................   1   0   JCK-1
...........................................................................................................................................................ucaaauccauuaaaauccauca..........................................................................   1   0   JCK-3
............................................................................................................................................................caaauccauuaaaauccauca..........................................................................   1   0   JCK-3
............................................................................................................................................................caaauccauuaaaauccauca..........................................................................   1   0   JCK-1
.................................................................................................................................................................ccauuaaaauccaucauuuu......................................................................   4   0   LCK-3
..................................................................................................................................................................................uuuuuaaaaucuuuuCaaauu....................................................   1   1   LT-3
.............................................................................................................................................................................................................uuuuuuaauaucauuaGauuu.........................   1   1   JCK-2
.............................................................................................................................................................................................................uuuuuuaauaucauuaGauuu.........................   2   1   JCK-3
```
